# Identification and functional analysis of a novel *LHX1* mutation associated with congenital absence of the uterus and vagina

**DOI:** 10.18632/oncotarget.14455

**Published:** 2017-01-02

**Authors:** Wei Zhang, Xueya Zhou, Liyang Liu, Ying Zhu, Chunmei Liu, Hong Pan, Qiong Xing, Jing Wang, Xi Wang, Xuegong Zhang, Yunxia Cao, Binbin Wang

**Affiliations:** ^1^ Reproductive Medicine Center, The First Affiliated Hospital of Anhui Medical University, Hefei, China; ^2^ Graduate School, Peking Union Medical College, Beijing, China; ^3^ Center for Genetics, National Research Institute for Family Planning, Beijing, China; ^4^ MOE Key Laboratory of Bioinformatics, Bioinformatics Division and Center for Synthetic and Systems Biology, TNLIST/Department of Automation, Tsinghua University, Beijing, China; ^5^ Department of Psychiatry and Centre for Genomic Sciences, Li KaShing Faculty of Medicine, The University of Hong Kong, Hong Kong SAR, China; ^6^ Department of Obstetrics and Gynecology, Peking Union Medical College Hospital, Beijing, China; ^7^ Department of Medical Genetics, School of Basic Medical Sciences, Capital Medical University, Beijing, China

**Keywords:** LHX1, congenital absence of the uterus and vagina, Müllerian duct abnormality, whole exome sequencing, transcriptional activity

## Abstract

Congenital absence of the uterus and vagina (CAUV) is the most extreme female Müllerian duct abnormality. Several researches proposed that genetic factors contributed to this disorder, whereas the precise genetic mechanism is far from full elucidation. Here, utilizing whole-exome sequencing (WES), we identified one novel missense mutation in *LHX1* (NM_005568: c.G1108A, p.A370T) in one of ten unrelated patients diagnosed with CAUV. This mutation was absent from public databases and our internal database. Through the luciferase reporter analysis, we found that the mutation could change the transcriptional activity of *LHX1* and its effect on the regulation of the downstream target gene *GSC*, which might be associated with urogenital system development. In short, we concluded that the *LHX1* may be a pathogenic gene of CAUV. Our results demonstrate the power of whole exome sequencing and gene prioritization approach as diagnostic tools in clinical practice that help make genetic diagnosis of CAUV.

## INTRODUCTION

Müllerian duct abnormality (MDA) is a complex and serious female reproductive tract malformation, which bring serious physical and psychological impact on patients [1]. Congenital absence of the uterus and vagina (CAUV), also known as Mayer-Rokitansky-Küster-Hauser (MRKH) syndrome, is the most extreme MDA, characterized by the congenital aplasia of the uterus and the upper part of the vagina in women with normal ovaries, karyotypes and secondary sexual characteristics [2, 3]. It is the second most frequent cause of primary amenorrhea after Turner syndrome in females, and the estimated prevalence of the disease was about 1 in 4000 to 5000 female newborns [4]. Approximately 50% of women with CAUV have concomitant congenital cardiac, renal, skeletal and hearing abnormalities [3]. Notably, most of the reported patients are sporadic while limited familial cases suggested the disease was transmitted as an autosomal recessive inheritance with reduced penetrance and variable expressivity or a polygenic and multifactorial pathogenesis [3].

The underlying etiology of CAUV remains unclear to date. Though the genetic basis has been studied, limited informative CAUV-affected families prevented the identification of any loci through conventional genetic linkage analysis [5]. Previous genetic studies based on targeted mutagenesis in the mouse delineated the genes that involved in initial Müllerian duct formation [6]. Candidate genes can be classified into three categories according to their involvement in the initial formation, differentiation, or regression of Müllerian duct, respectively, which include members of the *HOXA, WNT* gene families, *Emx2, Pax2, AMH* (*AMHR2*) and *LHX1* [7]. However, most mutations found in these candidate genes frequently showed contradicting evidence of replications. What's more, hardly any candidate genes or mutations were verified by functional analysis to enhance the understanding of how and why they influenced the development of Müllerian ducts.

By performing whole exome sequencing (WES) on a cohort of Chinese Han CAUV patients, we identified a novel missense mutation in *LHX1* as the prioritized causative gene of the malformation. Functional experiments confirmed our inference by showing the mutation could change the transcriptional activity of *LHX1*. This project might provide a new potential association between CAUV and *LHX1*.

## RESULTS

### Identification of a new *LHX1* variant associated with CAUV

By performing whole-exome sequencing, quality control statistics, strict filtering criteria and bioinformatic analysis in a cohort of CAUV patients, we identified a novel heterozygous missense variant in the *LHX1* gene (NM_005568: c.G1108A, p.A370T) in one patient. The patient was admitted to hospital due to primary amenorrhea at the age of 21. Clinical examinations using a transvaginal ultrasonography, hysteroscopy, laparoscopy showed the absence of the uterus and the upper part of vagina. She had normal kidney and 46XX karyotype. Candidate variants were prioritized based on their mutational effects, animal models and functions of affected genes. *LHX1* is the only candidate gene that has been associated with CAUV in human with recurrent mutations.

**Figure 1:**
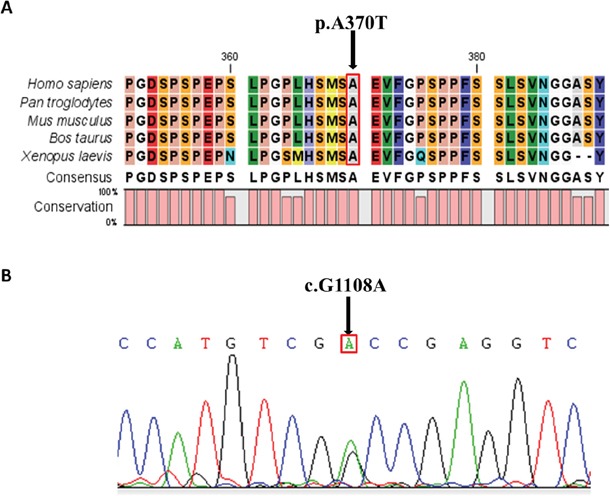
Conservative Prediction and Sequencing Validation **A.** Alignments of the amino acid sequence of part of LHX1 protein among different species. The mutation indicated by the arrow is in the conserved region of all the species shown (human, chimpanzee, mouse, cattle, African clawed frog); **B.** Validation of p.Ala370Thr (c.G1108A) heterozygous variant. Sanger sequencing results are shown.

The variant was absent from public databases (1000Genomes, ESP6500si, ExAC) and our internal database (98 MDA patients sequencing data). *In silico* analysis predicated that this variant would have a function in disease causing. The mutated amino acid at this site was highly conserved among different species, including human, chimpanzee, mouse, cattle and African clawed frog (Figure 1A), aligned by the CLC Free Workbench 4 software. The variant was confirmed by Sanger sequencing (Figure 1B).

### The variant decrease the transcriptional and biological activity of LHX1

**Figure 2:**
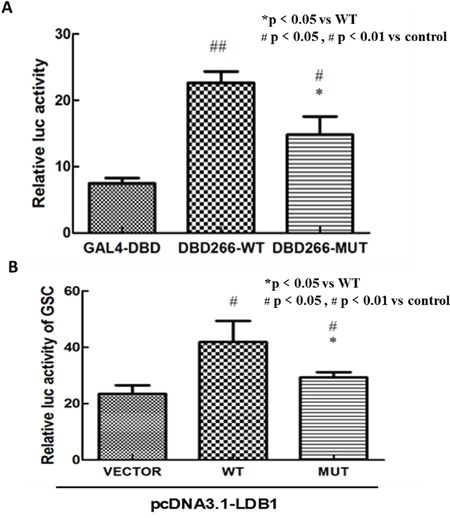
In vitro luciferase reporter assay **A.** Both wild-type and mutant pCMX-Gal4-DBD-CT266 plasmids had a significant increase in luciferase activity compared with pCMX-Gal4-DBD vector (p value<0.01, p value<0.05). The vector carrying this mutation showed a weakened luciferase activity compared with wild-type (p value<0.05). **B.** The luciferase activity of GSC-promoter reporter plasmid was significantly decreased in mutant pcDNA3.1-LHX1 group compared with wild group. Note: The results represent the mean (±SD) of 3 determinations in three independent experiments.

Luciferase reporter assay was used to evaluate whether the mutation affect the transcriptional or biological activity of *LHX1*. As the Figure 2A showed, both wild-type and mutant pCMX-Gal4-DBD-CT266 plasmids led to a significant increase in luciferase activity compared with pCMX-Gal4 empty vector (T test, *p<* 0.01; *p<* 0.05). However, the mutant group showed weakened luciferase activity compared with wild-type (T test, *p<* 0.05). These findings indicated that the mutation may have an inhibitory effect on the transcriptional activity of *LHX1*.

Using similar methods, we detected whether the variant affect the gene's ability of regulation of *GSC* transcription cooperate with *LDB1*. It turned out that the luciferase activity of *GSC*-promoter reporter plasmid was significantly decreased in mutant *LHX1* group compared with wild group (Figure 2B). These findings also indicated that the mutation may have an inhibitory effect on the ability of regulation of *GSC* transcription.

## DISCUSSION

*LHX1*, also known as *LIM1*, encodes a LIM-class homeodomain transcription factor that is essential for kidney and urogenital systems development [8]. Female *LHX1*-null mice present with normal ovaries but lack uterus, oviducts and the upper region of the vagina. Several human studies also confirmed its essential role in the development of the urogenital system [9, 10]. The recurrent 17q12 copy number losses was observed in 5~6% CAUV patients, which deleted one copy of *LHX1* genes [11, 12]. Sandbacka *et al*. supported the relevance of *LHX1* and copy number variations (CNVs) in the development of congenital malformation [13]. One missense single nucleotide variation (SNV) and a frameshift insertion of *LHX1* were found in two CAUV patients [7, 11]. From all of these researches, there is strong evidence that the haplo-insufficiency of *LHX1* is associated with CAUV.

The variant p.Ala370Thr we found in one CAUV patient was located in the transcriptional activation domain of carboxyl-terminal region of *LHX1. In vitro* luciferase reporter analysis indicated that the variant reduced the transcriptional activity of *LHX1* compared with the wild type. It was consistent with the findings that the amino acids region 266-403 of *LHX1*, in addition to homeodomains (178-238), is important for its transcriptional activation and interactions with other proteins [14]. *GSC* is a direct target gene for protein *LHX1* in the organizer and the prechordal plate of *Xenopus* [15]. Furthermore, it has been confirmed that microdeletion and homozygous predicted null mutations of *GSC* were involved in SAMS syndrome, which was characterized by unique rhizomelic skeletal anomalies, cryptorchidism (male), hypoplastic labia (female) and other urogenital anomalies [16]. So, we surmised that *LHX1* might cooperate with *GSC* to affect the development of urogenital system. Our results were consistent with this prediction as the activity of *GSC*-promoter reporter was significantly decreased in mutant *LHX1* group, cooperated with an important LIM-domain binding protein LDB1.

However, *LHX1* was previously sequenced in 96 Chinese MDA patients reported by Xia *et al*., but they did not find any missense or nonsense mutations [17]. Similarly, *LHX1* was also screened in 56 Caucasian MRKH syndrome patients and no mutation was identified [11]. Furthermore, in our project, rare missense SNV in *LHX1* did not seem to be enriched in 98 typical MDA patients. These findings illustrated that variants in *LHX1* may not be common genetic etiologic factors involved in female reproductive tract development malformations. More other pathogenic genes need to be found.

In summary, this is the first study to explore the pathogenic gene of CAUV by whole exome sequencing. In addition, we preliminary proposed the possible pathogenic mechanism of *LHX1* in CAUV, which provided a new evidence for the follow-up research, prevention and treatment of the disease. However, we hold the opinion that more systematic and intensive experiments are needed to explore the precise mechanism of the influence of *LHX1* on CAUV.

## MATERIALS AND METHODS

### Participants

This study included 10 Chinese Han unrelated CAUV patients that were recruited from Peking Union Medical College Hospital and the Fist Affiliated Hospital of Anhui Medical University. CAUV was diagnosed by experienced experts of gynecology and obstetrics according to medical history, results of karyotype analysis, androgen levels, and clinical examination using a transvaginal ultrasonography, hysteroscopy, laparoscopy and hysterosalpingogram.

This research was approved by the ethical committee of National Research Institute for Family Planning. The methods and experiments were carried out in accordance with approved guidelines. Informed written consents were obtained from all the participants.

### Whole-exome sequencing and validation

We sequenced exomes of the ten unrelated CAUV patients. All exons were captured by NimbleGen Human All Exon Enrichment kit and then massively parallel sequenced on Illumina Hiseq 2500 platform. We used BWA v0.5.9 [18] to align reads to reference human genome (GRC build 37), Picardtools to mark and remove duplicates, Samtools to detect SNPs and indels, and ANNOVAR [19] to annotate all variants. We only focused on functional variants (missense, nonsense, splicing site and frame-shift) with a minor allele frequency (MAF) less than 1% in public and in House databases. Frequency-filtered variants were then predicted *in silico* to assess their impact on the function and structure of encoded proteins. The analysis process referred to the article we published before [20]. The candidate gene detected by whole-exome sequencing was validated by Sanger sequencing.

### Site-directed mutagenesis and plasmids construction

The full CDS sequence of human *LHX1* (1221bp, 406 amino acids, NM_005568) was amplified by polymerase chain reaction (PCR) from cDNA. The variant p.A370T was constructed by site-directed mutagenesis using the Quick Change II Site-Directed Mutagenesis kit (Agilent Technologies, USA).

The wild and mutant truncated open reading frame (ORF) of human *LHX1*, CT266, were also amplified by PCR and inserted respectively into the pCMX-Gal4 vector [14]. The pCMX-Gal4 vector containing GAL4-DBD were provided by Dr. Ronald M. Evans (Salk Institute for Biological Studies, USA).

The wild and mutant *LHX1* were inserted respectively into the pcDNA3.1 vector (Invitrogen, Carlsbad, USA) to create the expression plasmid pcDNA3.1-*LHX1. LDB1* (NM_003893.4) was subcloned into the pcDNA3.1 vector as well. The promoter sequence of *goosecoid homeobox* (*GSC*) was amplified based on (http://switchdb.switchgeargenomics.com/productinfo/id_704725/), and then inserted into the luciferase reporter PLG3-basic vector.

### Transcriptional activity analysis and *in vitro* luciferase reporter assay

HEK 293 cells were seeded in 24-well tissue culture plates 18h prior to transfection (Lipofectamine 2000) at approximately 80% confluency. Cells were co-transfected with wild-type or mutant pCMX-Gal4-DBD-CT266 plasmids containing *LHX1* truncated ORF (300 ng/well) and the TK-promoter reporter plasmids (500 ng/well). The pCMX-Gal4-DBD vector was used as negative control. pREP7-RLu plasmid containing the *Renilla* luciferase gene cotransfection medium (10 ng/well) was used as an internal control. Thirty hours after transfection, cells were lysed with Passive Lysis Buffer (Promega, USA) and the Dual-luciferase Reporter Assay System (Promega) was used to assay the luciferase activity. Luminescence in relative light units (RLU) was measured for 10 s by luminescence detector (Promega). Firefly luciferase activity was first normalized to the level of *Renilla* luciferase activity. Results were then calculated as fold induction relative to pCMX-Gal4-DBD-CT266 wild-type vector.

HEK 293 cells were seeded in 24-well tissue culture plates as well. Luciferase assays plasmids including pcDNA3.1-*LDB1* (200 ng/well), pGL3-*GSC* promoter reporter vector (300 ng/well), and pcDNA3.1-*LHX1* (wild-type or mutant) or pcDNA3.1 empty vector (200 ng/well) were co-transfected into cells. Thirty hours after transfection, cells were treated the same way as above. Results were eventually calculated as fold induction relative to pcDNA3.1-*LHX1* wild-type vector as well.

Three independent experiments were performed in triplicate. The independent samples *T* test was adopted to determine statistical significance of unpaired samples. All data were analyzed by Excel (Microsoft 2010) and GraphPad Prism 5.

## Abbreviations

CAUV: Congenital absence of the uterus and vagina
WES: whole-exome sequencing
MDA: Müllerian duct abnormality
SNV: single nucleotide variation
MAF: minor allele frequency
PCR: polymerase chain reaction
ORF: open reading frame
